# SCNVSim: somatic copy number variation and structure variation simulator

**DOI:** 10.1186/s12859-015-0502-7

**Published:** 2015-02-28

**Authors:** Maochun Qin, Biao Liu, Jeffrey M Conroy, Carl D Morrison, Qiang Hu, Yubo Cheng, Mitsuko Murakami, Adekunle O Odunsi, Candace S Johnson, Lei Wei, Song Liu, Jianmin Wang

**Affiliations:** Department of Biostatistics and Bioinformatics, Roswell Park Cancer Institute, Buffalo, NY 14263 USA; Center for Personalized Medicine, Roswell Park Cancer Institute, Buffalo, NY 14263 USA; Department of Gynecologic Oncology, Roswell Park Cancer Institute, Buffalo, NY 14263 USA; Department of Pharmacology and Therapeutics, Roswell Park Cancer Institute, Buffalo, NY 14263 USA

## Abstract

**Background:**

Somatically acquired structure variations (SVs) and copy number variations (CNVs) can induce genetic changes that are directly related to tumor genesis. Somatic SV/CNV detection using next-generation sequencing (NGS) data still faces major challenges introduced by tumor sample characteristics, such as ploidy, heterogeneity, and purity. A simulated cancer genome with known SVs and CNVs can serve as a benchmark for evaluating the performance of existing somatic SV/CNV detection tools and developing new methods.

**Results:**

SCNVSim is a tool for simulating somatic CNVs and structure variations SVs. Other than multiple types of SV and CNV events, the tool is capable of simulating important features related to tumor samples including aneuploidy, heterogeneity and purity.

**Conclusions:**

SCNVSim generates the genomes of a cancer cell population with detailed information of copy number status, loss of heterozygosity (LOH), and event break points, which is essential for developing and evaluating somatic CNV and SV detection methods in cancer genomics studies.

## Background

Somatically acquired SVs and CNVs can introduce genetic changes that are directly related to tumor genesis [1,2]. SVs, including insertion, deletion, tandem duplication, inter- and intra-chromosome translocation, are changes of chromosome structure [3,4]. The size of a typical SV is usually greater than 1 kb. CNV, often regarded as a type of SV, was initially classified as gain or loss of a chromosome segment with a length greater than 1 kb, and then widened to include much smaller events (>50 bp) on accommodating the improved resolution of detection methods. Next-generation sequencing (NGS) has greatly improved the detection of somatic changes including SVs and CNVs [5,6]. A number of computational methods for detection of somatic SV/CNV have been developed [7,8]. However, accurate somatic SV detection for SVs mediated by long repeats, involving foreign insertion, or from minor clone in tumor cell population remains challenging. Similarly, factors such as tumor heterogeneity, purity, and aneuploidy impose major difficulties for somatic CNV detection [9].

A simulated cancer genome with known SVs and CNVs can serve as a benchmark for evaluating the performance of existing somatic SV/CNV detection tools and developing new methods. Currently, the SV/CNV simulations in literature mostly restrict to basic types such as insertions and deletions and often implement a known set of events (e.g., obtained from 1000 Genome Project) into the reference genome [10,11]. FUSIM is a sophisticated tool specialized on the simulation of fusion transcripts [12]. RSVSim is a more recent tool capable of simulating a wide ranges of SVs [13]. While they are excellent resource for simulating SV events in germline studies, they are not designed to simulate SV/CNV events in the context of commonly observed tumor sample characteristics such as aneuploidy, heterogeneity and purity. Moreover, B allele frequency (BAF) and LOH information, essential for CNV detection, are not provided by exiting tools.

Here, we describe a new simulation tool, SCNVSim, which focuses on generating a set of somatic SV and CNV events with caner related features such as tumor aneuploidy, heterogeneity and purity. The tool starts with the generation of a personalized genome with normal diploid status followed by simulation of somatic SVs and CNVs during tumor evolution.

## Implementation

**Figure 1:**
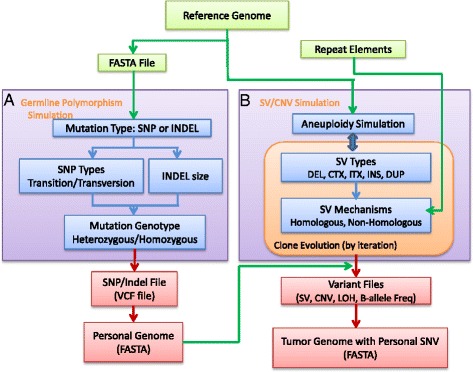
**The overall workflow of SCNVSim. A)** A personal genome with normal diploid status is generated by simulating SNV and INDEL against reference genome sequence. SNV/INDEL ratio, transition/transversion ratio, Heterozygous/Homozygous ratio and INDEL size distribution are considered (left). **B)** For tumor genome simulation, ploidy is first determined, followed by SV generations of different types and mechanisms (Non-homology or homology mediated). Heterogeneity is also implemented (right). The outputs include a simulated personal genome with normal diploid status in FASTA format, germline SNVs and INDELs in variant call format (VCF), and the following for each simulated tumor clone: 1) simulated SVs in terms of events and breakpoints, 2) copy number status for each individual segment, 3) BAF and LOH status of each segment, 4) FASTA format of cancer genome with somatic SV/CNV events as input for NGS reads simulation. By mixing the simulated genomes from the normal sample and tumor clones into a ratio specified by the user, a realistic and complicated cancer genome data set with varying levels of tumor heterogeneity and purity can be obtained.

As shown in Figure 1, SCNVsim consists of the following modules: 1) germline polymorphism simulation to generate a personal genome, 2) aneuploidy simulation to set the base ploidy, 3) SV/CNV simulation to generate different somatic events, 4) tumor heterogeneity simulation to generate multiple tumor clones, and 5) combining above simulations to generate complete tumor genomes with complex somatic SV and CNV events and varying levels of tumor heterogeneity and purity.

### Simulation of germline polymorphism

Somatic CNVs often demonstrate LOH which can be detected using BAF of heterozygous loci across the genome. Germline polymorphism, including SNVs (single nucleotide variations) and small INDELs (insertions and/or deletions which are smaller than 50 bp), provides such information and can be used in CNV detection [14]. SCNVSim simulates both SNVs and small INDELs with specified ratios of transition *vs.* transversion, heterozygous *vs.* homozygous, INDELs *vs.* SNVs, and distribution of INDEL size. The default setting are based on observations in publications [15-20], and all these parameters can be specified by users to change the behavior of the simulator and better serve a purpose for the user’s simulation. Combining the reference human genome (hg18, hg19 or hg38) with simulated germline SNV/INDELs, a personal genome with normal diploid status is obtained. BAF and LOH data can be obtained from the heterozygous SNVs and INDELs in the simulated personal genome.

### Simulation of tumor aneuploidy

Aneuploidy is a condition of abnormal number of chromosomes at the genome level. It is common in many cancer types and is a hallmark of chromosomal instability [21]. Aneuploidy is a major challenge for tumor CNV detection, as misidentification of base ploidy often causes the incorrect calling of gain or loss status. Aneuploidy simulation determines the base ploidy of the genome which can be specified by the users. The resulting aneuploidy chromosomes are randomly generated from the normal diploid genome and provide the starting genome for somatic SV simulation.

The exact aneuploidy status of each genome can be specified by users. For a monosomy genome (1n), one copy of the diploid chromosome is randomly deleted; for trisomy genome (3n), one copy of the diploid chromosome is randomly doubled; for tetrasomy (4n) or other even copy number of chromosomes, the normal genome is multiplied; and for pentasomy (5n) or odd copy number of chromosomes, the normal genome is multiplied first followed by random doubling of one extra copy of all chromosomes. By default, the functionality of large scale chromosome rearrangements is also implemented. Specifically, after aneuploidy simulation, a certain number of chromosomes will be randomly selected to generate whole or segmental chromosome duplications or deletions.

### Simulation of somatic SVs and CNVs

*Types* SCNVSim can simulate the following types of SV events: insertions, inversions, deletions, tandem duplications, inter- and intra-chromosomal translocations. Insertion is an event that occurs when the sequence of one or more nucleotides is added between two adjacent nucleotides in the genome. Inversion is an event that occurs when a continuous nucleotide sequence is inverted in the same position. Deletion is an event that occurs when a DNA segment is excised from the genome and the two nucleotides adjacent to the two ends of the excised segment fuse. Tandem duplication is a special insertion event, in which a DNA segment is copied, and then inserted to the position adjacent to itself. Inter-Chromosomal Translocation is an event that occurs when a region of nucleotide sequence is translocated to a new position in a different chromosome. Intra-Chromosomal Translocation is an event that occurs when a region of nucleotide sequence is translocated to a new position in the same chromosome with inverted orientation. Translocation could be balanced (no loss of genome) or unbalanced (loss of genome segment). The combinations of these events could lead to complex events of chromosomal rearrangement in cancer genome. Some of these types may cause CNVs such as deletions, tandem duplication and un-balanced translocations. The final copy number status of chromosomal segments is determined by properly calling tumor aneuploidy and copy number changing SV events.

**Figure 2:**
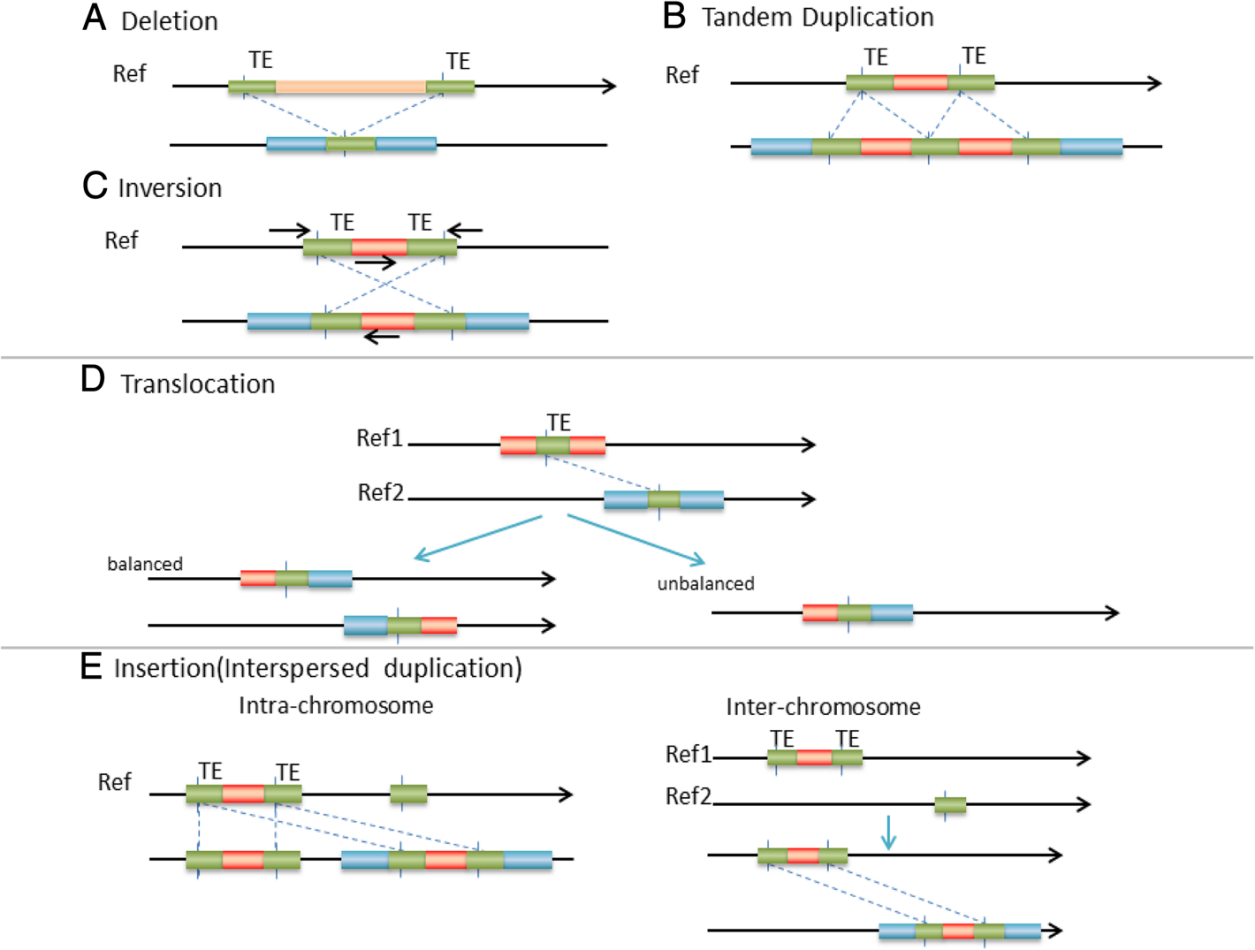
**Homologous sequence mediated SV simulation.** The types of homologous sequence (e.g., transposable elements) medicated SV simulated by SCNVSIM are: **A)** TE-mediated deletion; **B)** TE-mediated tandem duplication; **C)** TE-mediated inversion (two TEs are on opposite strands); **D)** TE-mediated translocation (balanced or unbalanced); and **E)** TE-mediated insertion (intra- or inter-chromosome). Break points are randomly picked in homologous sequences shared by compatible repeat elements which are from the same repeat family and overlapping with each other.

*Breakpoint simulation* Other than types, an important perspective of SV/CNV simulation is breakpoint information. Without loss of generosity, the breakpoints can be broadly classified into three different groups: breakpoint without homologous sequence, breakpoint with homologous sequence, and breakpoint with foreign insertion [22-25]. Non-homologous or micro-homologous breakpoints (<= 20 bps) are relatively easy to detect while homologous breakpoints could impose more challenges. SCNVSim simulates non-homologous breakpoints by randomly selecting breakpoints on the genome. For homologous breakpoints, SCNVSim utilizes the UCSC repeat mask database to identify genomic locations of repeat families (e.g., transposable elements (TE)). Repeat element mediating SVs require compatible elements, which are from the same repeat family and share homologous sequences. The types of TE mediated events are illustrated in Figure 2. Foreign insertion at a breakpoint is a relatively rare incident compared with the previous two groups. For SVs with this group of breakpoint, SNCVSim simulates novel (non-template) sequence that cannot be mapped to the reference genome but is inserted into the breakpoint.

### Simulation of tumor heterogeneity and purity

Tumor cell populations often display great heterogeneity with different sub-clones that evolve during tumor progression and treatment [26]. Such a mixture is one of the major obstacles for accurate SV/CNV identification in cancer genome studies. Tumor heterogeneity can be simulated by SCNVSim through clone evolution model [27], which hypothesizes that tumor starts from a founder clone and evolves into different sub-populations. First, an intermediate founder clone that has common SV/CNVs shared by all descendant clones is simulated. Then, several sub-clones are independently generated. By iterating this strategy, a more complicated tumor population can also be simulated. In addition, SCNVSim can simulate tumor heterogeneity through the cancer stem cell (CSC) model [28-30], which hypothesizes that only a small population of CSC is tumorigenic and tumor heterogeneity is due to the different ancestor CSC. As the different sub-clones in the CSC model do not necessary share common somatic SVs and CNVs, they can be obtained by running the independent SCNVSim simulation multiple times.

By coupling with NGS reads simulator and mixing the short reads from the aforementioned germline sample and tumor clones into a ratio specified by the user, a realistic and complicated cancer genome NGS data set with varying levels of tumor purity can be obtained for modeling different scenarios.

### Input, output and usage

SCNVSim takes a reference genome as input and outputs comprehensive information necessary for developing and evaluating somatic CNV and SV detection methods using NGS data.

*Input* When simulating germline polymorphism, SCNVSim takes chromosome length information and reference genome sequence file as the input. The inputs for somatic SV/CNV simulation include 1) the repeat mask file, 2) the germline SNV and INDEL file generated from germline simulation, 3) chromosome length file, and 4) the reference sequence file.

**Figure 3:**
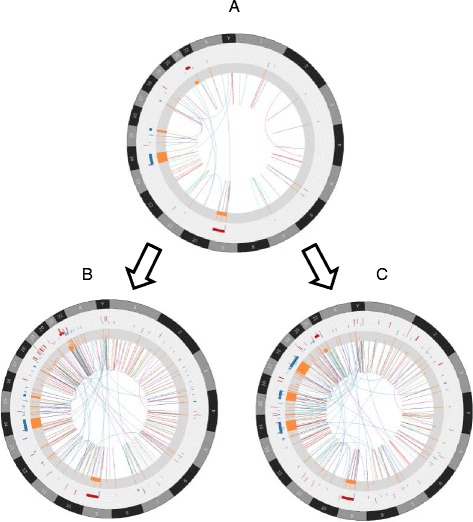
**The Circos plots of three simulated tumor clones. A)** The ancestor clone with 50 simulated SVs, **B)** the first descendant clone with 150 simulated SVs, and **C)** the second descendant clone with 150 simulated SVs. B and C are independently generated from A. For each Circos plot, the outer circle plots CNV with gain as red and loss as blue. The middle circle shows LOH status using orange. The inner circle shows SVs using the following color schema: inversion as red, insertion as blue, ITX as cyan, balanced CTX as magenta, and unbalanced CTX as brown.

*Output* The output of germline simulation includes a simulated personal genome with a normal diploid status in FASTA format and a file containing germline SNVs and INDELs in variant call format (VCF). The output of somatic SV/CNV simulation includes the following for each simulated tumor clone: 1) simulated SVs in terms of events and breakpoints, 2) copy number status for each individual segment, 3) BAF and LOH status of each segment, 4) FASTA format of simulated cancer genome with somatic SV/CNV events as input for NGS reads simulation tool [31]. As an example, we use SCNVSim to simulate 3 tumor clones with specified number of SV events under the clone evolution model. One ancestor clone (50 SV events) is generated first as the founder one, and then the other two clones (with 150 SV events each) are independently derived from the ancestor clone. The results are shown in Figure 3.

*Usage* A typical workflow for the SV/CNV algorithms assessment consists of SV/CNV event simulation followed by reads simulation. Once the FASTA-files with the simulated, rearranged cancer genome as well as simulated, normal germline genome are obtained from SCNVSim, they can be used as the input of a selected NGS read simulators (e.g., ART [31] to generate various NGS datasets for algorithm evaluation. A readme file with detailed descriptions of the functions, parameters and examples to combine SCNVSim with ART for tumor purity, heterogeneity, and aneuploidy simulation is included in the project homepage.

### Computing performance

We evaluated the computational efficiency of SCNVSim with different parameter settings, including the number of SV events, ploidy status and number of sub-clones, in both human and mouse reference genomes. The computing performances, including memory usage and runtime statistics, are recorded and summarized in Table 1.

**Table 1:** The CPU and memory usage for SCNVsim simulations with different parameter settings, including the number of SV events, ploidy status and number of sub-clones, in both human and mouse reference genomes*

**Simulation**	**Simulation parameters**	**Human (hg19)**		**Mouse (mm10)**	
		**CPU (min)**	**Memory (GB)**	**CPU (min)**	**Memory (GB)**
Germline simulation	Default parameters	3.9	7.9	3.3	7.3
Tumor simulation	single clone with 50 SVs	2.1	6.2	1.7	5.5
	single clone with 50 SVs, triploid	2.6	6.3	2.4	5.4
	single clone with 50 Svs, tetraploid	3.6	6.8	2.9	5.6
	single clone with 200 SVs	2.1	6.3	1.9	5.6
	single clone with 300 SVs	2.3	6.6	1.9	5.6
	2 clones with 50 and 150 SVs	3.9	7.9	3.6	6.4
	3 clones with 50, 150, and 150 SVs	5.9	8.0	4.7	7.1

*Analysis was performed on a Linux computer with two Intel® Xeon(R) E5-2620 v2 CPUs and 32 GB memory.

## Conclusions

Here we described a somatic CNV and SV simulator focusing on features related to cancer genome. It can simulate multiple types of SVs and CNVs in the context of tumor aneuploidy, tumor heterogeneity and tumor purity. By providing realistic cancer genomes as benchmarks, SCNVSim provides an alternative approach to evaluate the performance of SV/CNV detection algorithms and to help developers improve detection methods.

## Availability and requirements

**Project name:** SCNVSim

**Project home page:**http://sourceforge.net/projects/scnvsim

**Operating system(s):** Windows, Unix-like (Linux, Mac OSX)

**Programming language:** Java

**Any restrictions to use by non-academics:** None
